# Effects of whole-plant corn and hairy vetch (*Vicia villosa Roth*) mixture on silage quality and microbial communities

**DOI:** 10.5713/ab.23.0117

**Published:** 2023-06-26

**Authors:** Yaqian Zong, Kai Zhou, Xinhui Duan, Bo Han, Hua Jiang, Chenggang He

**Affiliations:** 1 Faculty of Animal Science and Technology, Yunnan Agricultural University, Kunming, Yunnan 650201, China

**Keywords:** Corn, Hairy Vetch, Microbial Communities, Silage Quality

## Abstract

**Objective:**

Hairy vetch is considered to improve the nutritional value of corn because of its high protein and mineral levels. To better understand the mechanism underlying hairy vetch regulated whole-plant corn silage fermentation, this experiment investigated the fermentation quality and bacterial community of whole-plant corn and hairy vetch mixture.

**Methods:**

Whole-plant corn and hairy vetch were mixed at ratios of 10:0 (Mix 10:0), 8:2 (Mix 8:2), 6:4 (Mix 6:4), 4:6 (Mix 4:6), 2:8 (Mix 2:8), and 0:10 (Mix 0:10) on a fresh weight basis. After ensiling 60 days, samples were collected to examine the fermentation dynamics, ensiling characteristics, and bacterial communities.

**Results:**

Mix 0:10, Mix 2:8, and Mix 4:6 showed poor fermentation characteristics. Mix 8:2 and Mix 6:4 silages showed high quality, based on the low pH, acetic acid, and ammonia nitrogen levels and the high lactic acid, crude protein, and crude fat contents. The bacterial diversity was affected by the mixing ratio of the two forage species. The genus *Lactobacillus* dominated the bacterial community in Mix 10:0 silage, whereas with the addition of hairy vetch, the relative abundance of *unclassified-Enterobacter* increased from 7.67% to 41.84%, and the abundance of *Lactobacillus* decreased from 50.66% to 13.76%.

**Conclusion:**

The silage quality of whole-plant corn can be improved with inclusion levels of hairy vetch from 20% to 40%.

## INTRODUCTION

Ensiling is commonly used in animal husbandry to preserve high-quality forage, and the quality of the silage is closely related to the chemical composition of the fresh material. Whole-plant corn (*Zea Mays* L) is an important feedstuff for ruminants because of its large biomass yield and high concentration of water-soluble carbohydrates (WSC) [1]. However, it has low protein content [2] and is therefore, in conventional practice, often mixed with high-protein forage crops for silage to improve its nutritional value. Hairy vetch (*Vicia villosa Roth*) can be found in southwest China, and it is a prolific and kind of annual or biennial legume grass that occupies an important position in animal feed herbage due to its good quality, high annual yield, lush stems and leaves, soft and succulent easy to cultivation, rich in nutrients, more than 20% crude protein (CP) content, and rich in vitamins and amino acids [3]. Hairy vetch has strong cold, drought, and barren soil tolerance and can be planted at higher altitudes. Under the condition of a shortage of protein feed resources in western China, the problem can be solved by planting hairy vetch leguminous grass. However, the direct ensiling of hairy vetch is challenging because of its high buffering capacity (BC) and low WSC content [4,5]. To achieve complementary silage characteristics, whole-plant corn could be mixed with hairy vetch for ensiling, which results in improved nutrient conservation and use of the silage. Previous studies have shown that whole-plant corn quality could be improved by mixing silage with high-protein leguminous forages [6,7].

Ensiling is a microbial driven process in which bacteria play an important role [8]. As the silage quality is affected by the bacterial community, monitoring the bacterial community and its succession could effectively promote silage fermentation [9]. Among the various mixed silages of whole-plant corn and legume forages, legume forages have been proposed as an effective stimulant to improve silage quality and change abundance of microbes [10,11]. Recently, some researchers found silage produced from the mixture of sweet corn stalks and lucerne effectively improved fermentation characteristics and bacterial community structure [10]. Similarly, He et al [11] reported that corn stalk and stylo with bauhinia variegate flower mixed silage can enhance the abundance of beneficial microbes. However, until now few publications relate to the microbial community and fermentation products during ensiling process of whole-plant corn and hairy vetch mixture silage.

Although it is generally acknowledged that mixing hairy vetch with whole-plant corn can improve the nutritional values of silage, the effects of different proportional ratios on silage quality and bacterial diversity are still largely unclear. It was speculated that a mixture of corn and hairy vetch could improve the silage quality, and that the relative abundance of the harmful bacteria involved in silage fermentation might increase with the increase of hairy vetch proportions. Therefore, the present study aimed to assess the effect of ensiling mixtures of whole-plant corn and hairy vetch under different mixing ratios on the fermentation dynamics, chemical composition, and bacterial diversity, which might provide important information for further regulation of fermentation.

## MATERIALS AND METHODS

### Raw materials and silage preparation

Corn and hairy vetch were cultivated in an experiment plot (latitude E 102°45′, N 25°8′) of Yunnan Agricultural University, Kunming, China. Both species were harvested on 26 November 2021. Fresh corn was manually harvested at the 1/3 maturity stage, with a stubble height of 10 cm, and fresh hairy vetch was collected at the blooming stage, with a stubble height of 5 cm. Before ensiling, corn, and hairy vetch were cut into pieces of 1 to 3 cm in length, divided into two parts (corn and hairy vetch), and blended at proportions of 100% whole-plant corn (Mix 10:0), 80% whole-plant corn + 20% hairy vetch (Mix 8:2), 60% whole-plant corn + 40% hairy vetch (Mix 6:4), 40% whole-plant corn + 60% hairy vetch (Mix 4:6), 20% whole-plant corn + 80% hairy vetch (Mix 2:8), and 100% hairy vetch (Mix 0:10) on a fresh matter (FM) basis. The fully mixed silage was packed into plastic drum silos (5-L volume, 18×25 cm; Yunnan Taineng Plastic Industry Co. Ltd, Kunming, China) and compressed manually, followed by sealing with a screw top. Each silo contained 2.5 kg of fresh material, and a total of 18 silos (6 mixing ratios×3 replicates) were fermented for 130 d at room temperature.

### Laboratory analyses

For analyses, 10 g of silage was mixed with 90 mL of sterilized water, and serially diluted. The diluted filtrate was plated on De Man, Rogosa, and Sharpe (MRS) agar (BS1137; Baisi Biotechnology Co., Ltd, Hangzhou, China), potato dextrose agar (BW010; Bio-way Technology Co., Ltd, Shanghai, China), and nutrient agar (HB0109; Hope Bio-Technology Co., Ltd, Qingdao, China) at 32°C for 72 h for enumeration of lactic acid bacteria (LAB), yeast, mold, and aerobic bacteria, respectively.

The silage extract was prepared using 20 g of sample diluted with 180 mL of distilled water in a juicer (Midea Group Co. Ltd., Guangdong, China) for 2 min. The homogenate was filtered through four layers of gauze, and the filtrate was immediately analyzed using a pH meter (ST310 ZH Ohouse Instruments Co., Ltd. Changzhou, China). Part of the extract was centrifuged (GL-20G-II Anting Scientific Instrument Factory, Shanghai, China) at 18,000×g for 15 min at 4°C. The supernatant was tested for ammonia nitrogen (NH_3_-N) and organic acids; NH_3_-N was analyzed referring to Broderick and Kang [12], and organic acids (lactic acid [LA], acetic acid [AA], propionic acid [PA], and butyric acid [BA]) were determined via high-performance liquid chromatography (HPLC, Ultimate 3000; Thermo Fisher Scientific, Waltham, MA, USA) [13]. The program execution was as set by Wang et al [14].

Fresh forage and silages were dried at 65°C to constant mass, analyzed for dry matter (DM) content, and then crushed through a 1-mm screen prior to chemical analysis. According to the method used by Van Soest et al [15], the neutral detergent fiber (NDF) with a heat-stable amylase and acid detergent fiber (ADF) of the silage were analyzed. The difference between NDF and ADF was used to calculate the hemicellulose content (Hemicellulose = NDF – ADF). The CP content was determined using an automatic Kjeldahl nitrogen determinator (K1100; Hanon Scientific Instruments Co., Ltd. Shandong, China). The WSC content was analyzed based on the anthrone-concentrated sulfuric acid method [16], and the ether extract (EE) content was determined according to Horii [17]. The BC was analyzed by the LA titration method [12].

### Bacterial community diversity analysis

#### DNA extraction

Briefly, 10 g silage was mixed with 90 mL of sterile water and shaken at 150 rpm for 15 min. The mixture was filtered through two layers of medical gauze and centrifuged at 10,000×g for 15 min at room temperature. The DNA was extracted from the silages using an E.Z.N.A. Soil DNA Kit (Omega Biotek, Norcross, GA, USA), following the manufacturer’s recommendations. The concentration of the DNA was measured using a Qubit 3.0 (Life Technologies, Carlsbad, CA, USA) to ensure that adequate amounts of high-quality genomic DNA had been extracted.

#### 16S rRNA gene amplification by polymerase chain reaction

The bacterial 16S rRNA V3-V4 variable regions were amplified using the universal primers 341F (5′-CCTACGGGNG GCWGCAG-3′) and 805R (5′-GACTACHVGGGTATCTA ATCC-3′). The polymerase chain reaction (PCR) amplification was performed under the following conditions: 95°C for 3 min, followed by 25 cycles of 95°C for 30 s, 55°C for 30 s, and 72°C for 30 s, with a final elongation step of 5 min at 72°C. The PCR products were checked using electrophoresis in 1% (w/v) agarose gels in TBE buffer (Tris, boric acid, EDTA) stained with ethidium bromide and visualized under UV light.

#### 16S gene library construction, quantification, and sequencing

Samples were delivered to Sangon BioTech (Shanghai, China) for library construction using a universal Illumina adaptor and index. Before sequencing, quality control was performed using a bioanalyzer (Agilent 2100, Santa Clara, CA, USA). Depending on the coverage requirements, all libraries can be pooled in a single run. Equimolar proportions were pooled according to the concentration of the purified amplicon. The PCR products were sequenced on an Illumina MiSeq platform (Illumina MiSeq, San Diego, CA, USA), following the manufacturer’s recommendations.

#### Sequence processing

Operational taxonomic units (OTUs) were clustered at a similarity level of 97% using Usearch (V11.0.667). All the software was in the mothur package. We submitted the effective sequences of each sample to the ribosome database project (RDP) Classifier again to identify archaeal and bacterial sequences. Species richness and diversity statistics including Coverage, Chao, Ace, Simpson, and Shannon ever were also calculated using mothur.

### Statistical analysis

All data were analyzed using the Statistical Package for the Social Sciences (SPSS Version 20.0; SPSS Inc., Chicago, IL, USA) via one-way analysis of variance. Polynomial contrasts were used to test linear and quadratic effects of the treatments to evaluate the effect of hairy vetch proportion on silage parameters. Significance was considered when p<0.05.

## RESULTS

### Chemical and microbial compositions of fresh raw materials

The chemical and microbial properties of the fresh material are shown in Table 1. The whole-plant corn was harvested at a DM of 300 g/kg FM, with a high WSC (105 g/kg DM) and a low CP (51.7 g/kg DM), whereas the DM, WSC, and CP levels of hairy vetch were 216 g/kg FM, 32.9 g/kg DM, and 202 g/kg DM, respectively. The DM and WSC contents of corn were higher than those of hairy vetch. Fresh hairy vetch had a high BC (70.7 mEq/kg DM) and the number of surface LAB was below 4.54 log_10_ cfu/g of FM, indicating weak fermentation ability. The content of DM and WSC gradually decreased as the proportion of hairy vetch in raw materials increased and the content of DM and WSC in corn, Mix 8:2 and Mix 6:4 groups were significantly higher than that in the other three groups (p<0.01). Besides, adding hairy vetch had also significantly reduced the LAB numbers in the silage (p<0.01). On the contrary, the contents of CP and EE increased (p<0.01) with increasing inclusion levels of hairy vetch.

### Fermentation parameters, chemical and microbial composition of the silage

The fermentation parameters of the mixed silage are shown in Table 2. By comparing the fermentation parameters of corn mixed with hairy vetch, the pH value, the NH_3_-N, concentration of LA and AA were dramatically influenced by the mixing ratio of raw material. The data support the following observations, the pH value and the NH_3_-N content increased with increasing inclusion levels of hairy vetch. The Mix 10:0 silage had the lowest pH (3.95) and NH_3_-N (20.9 g/kg) values among all silages (p<0.05). The concentration of LA in the Mix 10:0 (81.0 g/kg DM) group was significantly higher than that in the Mix 8:2 (69.9 g/kg DM), Mix 4:6 (65.2 g/kg DM), Mix 2:8 (54.9 g/kg DM) and Mix 0:10 (21.4 g/kg DM) groups (p<0.01), while the AA concentration was significantly lower than that in the Mix 8:2, Mix 6:4, Mix 4:6, Mix 2:8 and Mix 0:10 groups (p<0.01). Free of PA in all mixed silage samples. In general, Mix 0:10 silage was slightly fermented, based on the lowest LA (21.4 g/kg DM) content and the highest pH (5.11), BA (5.48 g/kg DM), and NH_3_-N (83.2 g/kg) levels.

As shown in Table 3, the DM values of Mix 6:4 (267 g/kg FM), Mix 4:6 (252 g/kg FM), Mix 2:8 (229 g/kg FM), and Mix 0:10 (184 g/kg FM) silages were significantly lower (p<0.01) compared to those of Mix 10:0 (286 g/kg FM) and Mix 8:2 (272 g/kg FM) silages. The content of WSC gradually decreased as the proportion of hairy vetch in raw materials increased and the content of WSC in Mix 2:8 and Mix 0:10 groups were significantly lower than that in the other four groups (p<0.01). The contents of CP and EE increased (p< 0.01) with increasing inclusion levels of hairy vetch; compared with Mix 10:0, the levels in Mix 0:10 were 3.26 and 2.61 times higher, respectively. Adding hairy vetch had no effects on ADF but significantly reduced the NDF values in the silage (p<0.01).

The microbial numbers of the mixed silage are shown in Table 4. The number of LAB in mixed silage decreased with the proportion of hairy vetch increasing. On the contrary, there existed increasing trends in counts of aerobic bacteria following the increase of the proportion of hairy vetch in mixed silage. Yeast was undetectable in the Mix 4:6 and Mix 2:8 silages and the highest yeast count was found in Mix 0:10 silage. The mold was not detected in all silages.

### Bacterial diversity of the silage

After discarding unqualified sequences, the reads ranged from 40,811 to 45,824 and were clustered into a total of 1,363 OTUs. As shown in Table 5, all the samples of bacteria were sequenced as indicated by coverage of >99.9, indicating that the identified sequences represented the majority of micro-biota in silage. Additive hairy vetch mixed silage to corn affected the OTU number, Ace, Chao, Shannon, and Simpson indices of bacterial diversity. The results of mixed silage of corn and hairy vetch showed that the OTU number, Ace index, and Chao index first increased and then decreased as the proportion of hairy vetch in raw materials increased, Mix 6:4 had the highest OTU number, Ace index, and Chao index. For community richness comparison, the Shannon index was lower, and the Simpson index was higher in the Mix 0:10 compared with the Mix 10:0 group. The Shannon index of corn single storage was 2.29, those of corn and hairy vetch mixtures 2.30 to 3.17, and that of hairy vetch single storage was 2.00.

**Figure 1:**
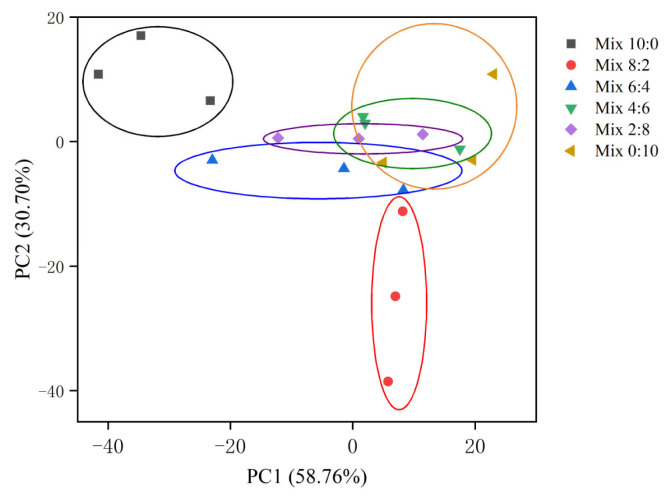
Principal component analysis of corn and hairy vetch mixed silage bacterial community. Corn and hairy vetch were mixed at proportions of 10:0 (Mix 10:0), 8:2 (Mix 8:2), 6:4 (Mix 6:4), 4:6 (Mix 4:6), 2:8 (Mix 2:8), and 0:10 (Mix 0:10) on a fresh weight basis, respectively.

Principal components analysis (PCA; Figure 1) showed differences among the six groups in terms of bacterial communities, where components 1 (PC1) and 2 (PC2) expressed 58.76% and 30.70% of the total variance, respectively. Mix 10:0 silage samples were separated from the samples treated with addition of hairy vetch, indicating that the addition of hairy vetch influenced the bacterial community.

### Comparisons at the genus level

**Figure 2:**
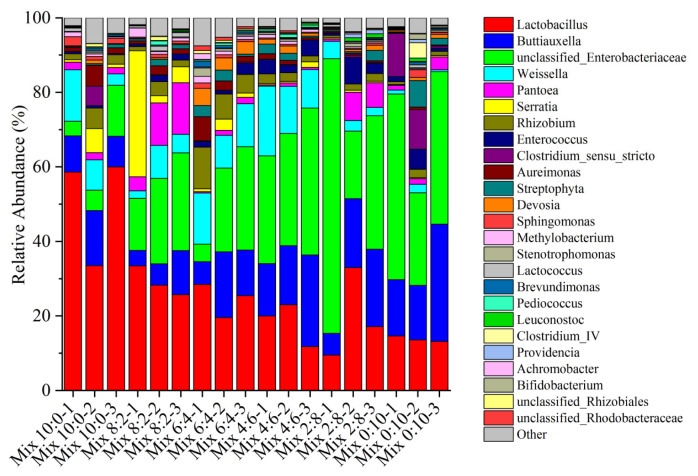
The bacterial community and relative abundance of mixed silage of corn and hairy vetch in different proportions. Corn and hairy vetch were mixed at proportions of 10:0 (Mix 10:0), 8:2 (Mix 8:2), 6:4 (Mix 6:4), 4:6 (Mix 4:6), 2:8 (Mix 2:8), and 0:10 (Mix 0:10) on a fresh weight basis, respectively.

Figure 2 shows the main bacterial communities of corn and hairy vetch mix silage at the genus level. The dominant bacterial genera in the silage samples were *Lactobacillus*, *Buttiauxella*, *Weissella*, *Pantoea*, *Serratia*, *Rhizobium*, and *unclassified Enterobacteriaceae*. The dominant bacteria in the six treatment groups were *Lactobacillus*, *Buttiauxella*, and *unclassified-Enterobacteriaceae*. *Lactobacillus* (50.66%) was the dominant genus in Mix 10:0, with the highest relative abundance. However, the abundances of *Buttiauxella* and *unclassified-Enterobacteriaceae* increased significantly after the addition of hairy vetch, and these genera were the dominant genera in Mix 0:10, Mix 2:8, and Mix 4:6 silages. The Mix 0:10 silage abundance of the undesirable *Clostridium-sensu-stricto* (7.59%) was higher when compared to those of the other silages. *Serratia* was mainly found in Mix 10:0 and Mix 8:2 silages, with abundances ranging from 2.69% to 3.11% of the total population. *Pantoea* (9.66%) and *Rhizobium* (7.64%) were mainly found in Mix 8:2 and Mix 6:4 silages.

### Correlation between bacterial abundance and fermentation parameters

**Figure 3:**
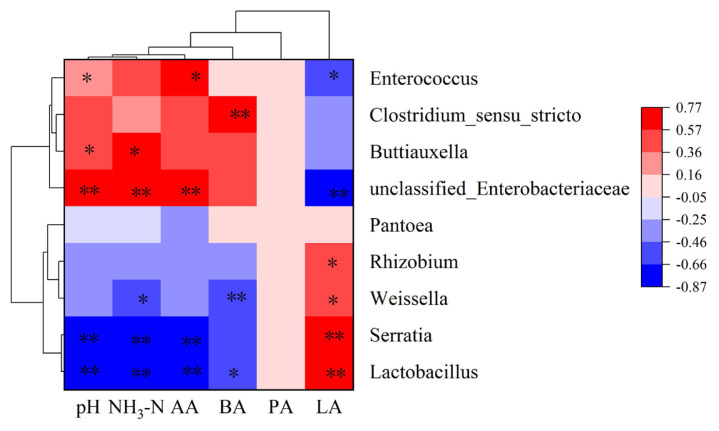
Correlation analysis between bacterial community and organic acid, pH and ammonia nitrogen; LA, lactic acid content; AA, acetic acid content; PA, propionic acid content; BA, butyric acid; NH_3_-N, ammonia-N. The corresponding value of the heat map is the spearman correlation coefficient r (–0.87 to 0.77); * p<0.05, ** p<0.01.

Figure 3 shows the relationships between bacterial diversity and fermentation parameters. The genera *Lactobacillus*, and *Serratia* were significantly negatively correlated with pH, whereas *unclassified-Enterobacteriaceae*, *Buttiauxella*, and *Enterococcus* were significantly positively correlated with pH (p<0.05). The genera *Lactobacillus*, *Serratia*, and *Weissell* were significantly negatively correlated with NH_3_-N, whereas *unclassified-Enterobacteriaceae*, and *Buttiauxella* were significantly positively correlated with NH_3_-N. The genera *Lactobacillus*, and *Serratia* were significantly negatively correlated with AA, whereas *unclassified-Enterobacteriaceae*, and *Enterococcus* were significantly positively correlated with AA content (p<0.05). The BA content was positively associated with genera *Clostridium-sensu-stricto* (p<0.01), while it was negatively associated with genera *Weissella*, and *Lactobacillus* (p<0.05). The genera *Lactobacillus*, *Weissella*, *Serratia*, and *Rhizobium* were positively associated with the contents of LA (p<0.05), and there was a negative correlation (p<0.05) between *unclassified-Enterobacteriaceae*, and *Enterococcus* and the LA content (p<0.05).

## DISCUSSION

### Chemical and microbial compositions of fresh raw materials

The chemical composition of fresh forage, particularly the WSC content, is a key parameter of LA fermentation [18]. Generally, the WSC content greater than 5% DM is an important condition for high-quality fermentation [19,20]. The WSC content in hairy vetch was 32.9 g/kg, which did not meet the minimum requirement for well-conserved silage. However, fresh hairy vetch has low DM (216 g/kg FM) contents and high BC (70.7 mEq/kg DM), which can cause clostridia fermentation and nutrient loss. Some previous studies have confirmed that legume forages ensiled alone cannot achieve high fermentation quality [14]. Studies have revealed that mixing the grass family, such as corn, with the legume family, helps improve fermentation quality and nutritional composition [21]. As the major raw material of ruminant feedstuff in China, corn has WSC (105 g/kg DM) and DM (300 g/kg FM) sufficiently, to make sure the fermentation is complete. The CP content of hairy vetch was 202 g/kg DM which is much higher than that of corn. According to the properties of the raw materials, the combination of the high WSC and DM contents in corn with the high protein levels in hairy vetch can be used to improve the nutritional value of the fresh materials.

According to a previous finding, the diversity and abundance of epiphytic microorganisms in the raw material are related to silage quality [22]. For example, in a study by Oliveira et al [23], when the LAB number exceeded 10^5^ cfu/g, LA fermentation can start rapidly; at levels below 10^5^ cfu/g, DM loss, and NH_3_-N concentration are likely to be higher. In our study, the surface LAB numbers of fresh corn (6.55 log_10_ cfu/g of FM) were higher than those of fresh hairy vetch (4.54 log_10_cfu/g of FM), which leads us to infer that corn is more susceptible to LA fermentation. Thus, hairy vetch provides more nutrients, while corn provides LAB and WSC when used in combination.

### Effects of mixed silage on fermentation quality, chemical composition, and bacterial numbers

The pH, NH_3_-N, and organic acids are important parameters to measure the quality of the fermentation and the microbial activity. Of these, pH is an important indicator of silage quality [24], and ideally, it should reach a level below 4.2 to provide a stable acidic environment [25]. In this study, the pH values of the Mix 10:0, Mix 8:2, and Mix 6:4 groups were close to or below 4.2, which could ensure good preservation of mixture silage. In contrast, Mix 0:10 has a higher pH value, even higher than 5.0 and such discrepancy may be attributed to different application rates of hairy vetch. Our results agree with those of Kung et al [26], who found that corn silages generally have lower final pH values than legume silages.

The NH_3_-N content represents the CP decomposition in silage, which indicates the extent of proteolysis in the silage [27]. The accumulation of NH_3_-N in the silage results from the combined action of plant protease activity and microbial metabolism [28]. The comparatively low NH_3_-N content in Mix 10:0, Mix 8:2 and Mix 6:4 silages could be attributed to the effect of lower pH values, indicating that the activity of protease was inhibited leading to the preservation of more nutrients. Wang et al [6] reported that the level of NH_3_-N increased with increasing inclusion levels of alfalfa, which was consistent with the experimental results obtained here. This can be explained by the fact that LAB has low activity, allowing undesirable bacteria to thrive at high pH levels and to degrade proteins to ammonia.

Organic acids in silage are the end products of various microbial activities [29]. The addition of hairy vetch increased the pH values of Mix 4:6, Mix 2:8 and Mix 0:10, which could promote harmful microorganisms and inhibit the reproduction and growth of LAB, leading to decreased LA content. However, different addition levels have various effects. In this experiment, an increased proportion of hairy vetch was accompanied by increased AA content, possibly because the sugar level in hairy vetch is low. Under the condition of insufficient sugar content, the silage mixture tends to transition from homo-fermentation to hetero-fermentation, which produces not only LA but also AA, ethanol, and CO_2_ [30]. Meanwhile, the AA is the end product of the activities of *Enterobacter*, *Acetobacter*, or the hetero-fermentative *Lactobacillus* [31]. These reasons together explain the higher AA content in Mix 0:10, Mix 2:8, and Mix 4:6 groups. In addition, the relative abundance of LAB decreased with increasing hairy vetch content, because the attached surface of hairy vetch has a low abundance of LAB, resulting in less LA content and a large number of harmful microorganisms. The presence of BA in silage is undesirable because it is caused by the fermentation of the undesirable genus *Clostridium*, and the BA level more than 5 g/kg DM indicates a significant reduction in livestock feed intake [30,32].

The chemical composition of the silages significantly differed among the different mixing ratios. In this experiment, the DM contents of Mix 10:0 (286 g/kg FM) and Mix 8:2 (272 g/kg FM) silages were relatively high (p<0.05), which was related to the proportion of corn in the mixed silage. The increases in CP and EE content were related to the proportion of fresh hairy vetch. In a similar study, Ozturk et al [33] found that the CP content increased with increasing inclusion levels of alfalfa in mixed silage of alfalfa and whole wheat. It has been reported that to ensure the normal activities of rumen microorganisms, the dietary CP content requires more than 70 g/kg DM, and low CP content may reduce the proliferation of rumen microorganisms [34]. In this study, the requirement of rumen microbial proliferation could be satisfied if the proportion of hairy vetch exceeded 20%, which was attributed to the abundant protein in it. The ADF and NDF are negatively correlated with digestibility and feed intake, respectively. The lower the values of ADF and NDF, the higher the digestibility and feed intake of the feed [35,36]. Here, the NDF level was significantly lower for Mix 0:10, Mix 2:8, and Mix 4:6 than for Mix 10:0, Mix 8:2, and Mix 6:4. Apparently, considerable loss of NDF occurred in mixed silage. This loss could be due to a combination of enzymatic and acid hydrolysis of the more digestible cell wall fractions during the fermentation. The decrease in the NDF content in the mixed silage was consistent with the experimental results reported by He et al [37]. The lowest LAB number and highest yeast counts indicated that hairy vetch poor fermentation quality. Moreover, the undesirable yeast and mold could be inhibited by abundant LAB and the low pH, leading to ensuring good fermentation. These results suggested that a combination of corn and hairy vetch could improve the feeding value of corn to some extent.

### Effects of mixed silage of corn and hairy vetch on bacterial communities

The Chao and Shannon indices are common indices reflecting microbial richness and diversity, respectively. In our study, the Mix 6:4 group showed the high OTU, Ace, Chao, and Shannon indices, as well as the low Simpson index, indicating a higher bacterial diversity. These results implied that an additive of 40% hairy vetch to corn for mixed silage could increase bacterial diversity. It could be explained that the addition of 40% hairy vetch to corn for mixed silage made the pH drop of silage slow and failed to immediately inhibit the activity of acid-labile microorganisms and inhibited the growth of LAB species. Therefore, the relative abundance of LAB decreased, leading to an increase in microbial diversity. Previous studies have shown that when LAB become the dominant species, they compete with a variety of microorganisms, thereby decreasing bacterial diversity [31,32].

Since bacteria are responsible for silage fermentation, exploring the bacterial community and relative abundances can improve our understanding of the fermentation levels in silage. The relative abundances of the 16 most microbes further explained the differences in the bacterial communities in mixed corn and hairy vetch at the genus level. *Lactobacillus* and *unclassified-Enterobacteriaceae* were the dominant bacteria in the six types of mixed silages, which agree with previous findings [29]. *Lactobacillus* increases the LA concentration while creating an acidic environment during silage fermentation [38]. The increase in hairy vetch was accompanied by a drop in the relative abundance of *Lactobacillus*, while the relative abundances of *Buttiauxella* and *unclassified-Enterobacteriaceae* were increased. Similarly, Yan et al [39] reported that mixed silage of corn and ryegrass significantly changed the bacterial community and influenced silage fermentation. It could be attributed that the addition of hairy vetch reduces the substrates and energy for *Lactobacillus*, which was not conducive to its propagation. In addition, the weakly acidic environment created by the addition of vetch also promotes the growth of undesirable microorganisms, which might inhibit the growth of *Lactobacillus*. *Weissella* is also widely distributed in silage, and most of its species are mostly obligate hetero-fermentative bacteria. Its metabolites are mainly LA and AA, which together with *Lactobacillus* determine silage quality [30]. Furthermore, the genera *Lactobacillus* and *Weissella* positively correlated with the LA content, suggesting that the increase in LA was mainly caused by *Lactobacillus* and *Weissella* metabolism. Similar to our findings, Guan et al [40] studies have reported that the genera *Lactobacillus* and *Weissella* are positively correlated with the concentration of LA silage. *Enterobacter* can not only convert LA into AA and other organic acids but can also metabolize protein to NH_3_-N, resulting in poor silage fermentation [41]. Mix 4:6, Mix 2:8 and Mix 0:10 increased the abundance of *Enterobacteria*, impeding the production of high-quality silage. Some studies have demonstrated that microbial metabolism would result in nutrient consumption during silage. Fermentation of *Clostridium* leads to the breakdown of protein, it can ferment LA or glucose to BA under anaerobic conditions and *Enterobacter* and *Lactobacillus* also compete for the limited nutrient composition, so *Clostridium* and *Enterobacter* were not conducive to maintaining the fermentation quality and nutrient of mixed silage. This relationship was also confirmed through the correlation of bacterial communities and organic acids. In conclusion, the bacterial communities affected silage quality by affecting fermentation quality.

## CONCLUSION

The content of hairy vetch could improve the silage quality of corn to different extents, but the fermentation quality of silage with the content of 20% and 40% is better than other treatments. The inclusion of 20% or 40% hairy vetch had higher LA content and raised contents of AA, pH value, and NH_3_-N in silage, putting it into the category of good fermentation. The inclusion of the 20% or 40% hairy vetch group also changed the bacterial community of silage, which increased bacterial diversity. However, the relative abundance of *Lactobacillus* and *Weissella*, in these two treatments was relatively high and the relative abundance of undesirable microorganisms was low. Besides, the inclusion of 20% or 40% hairy vetch can considerably improve silage quality, evidenced by an increase in CP and EE contents. In summary, the addition of 20% or 40% hairy vetch to corn can improve the nutritional quality of the silage.

## Figures and Tables

**Table 1:** Chemical and microbial properties of corn and hairy vetch prior to ensiling

Item	Treatment^1)^						SEM	p-value^2)^		
	
	Corn	Mix 8:2	Mix 6:4	Mix 4:6	Mix 2:8	Hairy vetch		T	L	Q
Chemical composition										
DM (g/kg FM)	300^a^	283^ab^	266^b^	248^c^	232^cd^	216^d^	7.24	<0.01	<0.01	0.87
WSC (g/kg DM)	105^a^	90.1^b^	77.5^b^	61.2^c^	47.4^d^	32.9^e^	6.76	<0.01	<0.01	0.20
CP (g/kg DM)	51.7^f^	77.7^e^	94.0^d^	132^c^	162^b^	202^a^	11.7	<0.01	<0.01	<0.01
NDF (g/kg DM)	522^a^	521^a^	500^a^	508^a^	539^a^	569^a^	31.4	0.51	0.65	0.40
ADF g/kg DM	242^a^	240^a^	253^a^	230^a^	238^a^	243^a^	4.73	0.88	0.83	0.91
Hemicellulose	280^a^	286^a^	247^a^	278^a^	301^a^	327^a^	11.7	0.57	0.24	0.23
EE (g/kg DM)	14.3^d^	16.1^d^	17.2^cd^	21.4^bc^	24.0^ab^	26.4^a^	1.74	<0.01	<0.01	0.53
BC (mEq/kg DM)	34.7^f^	43.6^e^	48.4^d^	55.0^c^	65.5^b^	70.7^a^	3.02	<0.01	<0.01	0.65
Microorganism log_10_ cfu/g of FM										
LAB	6.55^a^	6.11^b^	5.61^c^	5.34^d^	4.91^d^	4.54^e^	0.17	<0.01	<0.01	0.12
Aerobic bacteria	5.68^a^	5.60^a^	5.46^a^	5.60^a^	5.66^a^	5.65^a^	0.03	0.44	0.79	0.15
Yeast	5.28^a^	5.10^a^	4.83^b^	4.59^c^	4.60^c^	4.32^d^	0.08	<0.01	<0.01	0.37
Mold	4.65^c^	4.97^bc^	5.09^abc^	5.18^ab^	5.49^ab^	5.52	0.09	0.02	<0.01	0.66

SEM, standard error of the means; DM, dry matter; FM, fresh matter; WSC, water-soluble carbohydrates; CP, crude protein; NDF, neutral detergent fiber assayed with a heat stable amylase and expressed inclusive of residual ash; ADF, acid detergent fiber expressed inclusive of residual ash; EE, ether extract; BC, buffering capacity; LAB, lactic acid bacteria; CFU, colony-forming units.

1) Corn and hairy vetch were mixed at proportions of 8:2 (Mix 8:2), 6:4 (Mix 6:4), 4:6 (Mix 4:6), and 2:8 (Mix 2:8) on a fresh matter basis, respectively.

2) T, effect of treatment; L, linear effect of treatment; Q, quadratic effect of treatment.

a–f Means in the same row with different letters are significantly different (p<0.05).

**Table 2:** Fermentation parameter of the mixed corn and hairy vetch silage

Item	Treatment^1)^						SEM	p-value^2)^		
	
	Mix 10:0	Mix 8:2	Mix 6:4	Mix 4:6	Mix 2:8	Mix 0:10		T	L	Q
pH	3.95^e^	4.10^d^	4.24^c^	4.34^b^	4.68^b^	5.11^a^	0.94	<0.01	<0.01	<0.01
NH_3_-N (g/kg)	20.9^f^	32.5^e^	42.9^d^	51.8^c^	67.5^b^	83.2^a^	0.27	<0.01	<0.01	<0.01
LA (g/kg DM)	81.0^a^	69.9^b^	78.7^a^	65.2^c^	54.9^d^	21.4^e^	1.05	<0.01	<0.01	<0.01
AA (g/kg DM)	13.2^d^	14.5^c^	15.7^c^	25.9^b^	25.7^b^	33.8^a^	2.27	<0.01	<0.01	<0.01
PA (g/kg DM)	ND	ND	ND	ND	ND	ND	-	-	-	-
BA (g/kg DM)	ND	ND	ND	ND	ND	5.48	-	-	-	-

SEM, standard error of the means; NH_3_-N, ammonia-N; LA, lactic acid; AA, acetic acid; PA, propionic acid; BA, butyric acid; ND, not detected.

1) Corn and hairy vetch were mixed at proportions of 10:0 (Mix 10:0), 8:2 (Mix 8:2), 6:4 (Mix 6:4), 4:6 (Mix 4:6), 2:8 (Mix 2:8) and 0:10 (Mix 0:10) on a fresh matter basis, respectively.

2) T, effect of treatment; L, linear effect of treatment; Q, quadratic effect of treatment.

a–f Means in the same row with different letters are significantly different (p<0.05).

**Table 3:** Chemical composition of mixed corn and hairy vetch silage

Item	Treatment^1)^						SEM	p-value^2)^		
	
	Mix 10:0	Mix 8:2	Mix 6:4	Mix 4:6	Mix 2:8	Mix 0:10		T	L	Q
DM (g/kg FM)	286^a^	272^a^	267^b^	252^c^	229^d^	184^e^	1.06	<0.01	<0.01	<0.01
WSC (g/kg DM)	62.4^a^	47.1^c^	39.7^d^	35.3^d^	21.3^e^	18.6^e^	3.72	<0.01	<0.01	0.09
CP (g/kg DM)	56.1^f^	75.1^e^	97.3^d^	127^c^	156^b^	183^a^	1.08	<0.01	<0.01	0.04
NDF (g/kg DM)	612^a^	604^a^	560^b^	485^c^	468^c^	465^c^	1.55	<0.01	<0.01	0.27
ADF (g/kg DM)	315^a^	308^a^	298^a^	282^a^	278^a^	261^a^	0.98	0.69	0.14	0.80
Hemicellulose	297^a^	296^a^	262^ab^	203^b^	190^b^	200^b^	1.41	<0.01	<0.01	0.43
EE (g/kg DM)	12.7^c^	15.9^c^	22.8^b^	28.9^ab^	26.7^b^	33.1^a^	1.85	<0.01	<0.01	0.28

SEM, standard error of the means; DM, dry matter; FM, fresh matter; WSC, water-soluble carbohydrates; CP, crude protein; NDF, neutral detergent fiber; ADF, acid detergent fiber expressed inclusive of residual ash; EE, ether extract.

1) Corn and hairy vetch were mixed at proportions of 10:0 (Mix 10:0), 8:2 (Mix 8:2), 6:4 (Mix 6:4), 4:6 (Mix 4:6), 2:8 (Mix 2:8) and 0:10 (Mix 0:10) on a fresh weight basis, respectively.

2) T, effect of treatment; L, linear effect of treatment; Q, quadratic effect of treatment.

a–f Means in the same row with different letters are significantly different (p<0.05).

**Table 4:** Microbial numbers of mixed corn and hairy vetch silage

Item	Treatment^1)^						SEM	p-value^2)^		
	
	Mix 10:0	Mix 8:2	Mix 6:4	Mix 4:6	Mix 2:8	Mix 0:10		T	L	Q
LAB (log_10_ cfu/g of FM)	7.12^a^	6.91^b^	6.73^c^	6.29^d^	6.29^d^	5.80^e^	0.08	<0.01	0.04	0.17
Aerobic bacteria (log_10_ cfu/g of FM)	5.56^d^	5.87^c^	6.19^b^	7.12^a^	7.17^a^	7.29^a^	0.08	<0.01	<0.01	<0.01
Yeast (log_10_ cfu/g of FM)	1.78^b^	1.26^c^	1.04^c^	ND	ND	3.23^a^	0.10	<0.01	<0.01	<0.01
Mold (log_10_ cfu/g of FM)	ND	ND	ND	ND	ND	ND	-	-	-	-

SEM, standard error of the means; LAB, lactic acid bacteria; CFU, colony-forming units; FM, fresh matter; ND, not detected.

1) Corn and hairy vetch were mixed at proportions of 10:0 (Mix 10:0), 8:2 (Mix 8:2), 6:4 (Mix 6:4), 4:6 (Mix 4:6), 2:8 (Mix 2:8) and 0:10 (Mix 0:10) on a fresh weight basis, respectively.

2) T, effect of treatment; L, linear effect of treatment; Q, quadratic effect of treatment.

a–e Means in the same row with different letters are significantly different (p<0.05).

**Table 5:** Statistics of bacterial community diversity

Item	Treatment^1)^						SEM	p-value^2)^		
	
	Mix 10:0	Mix 8:2	Mix 6:4	Mix 4:6	Mix 2:8	Mix 0:10		T	L	Q
Read	40,811^a^	43,731^a^	44,357^a^	45,288^a^	45,824^a^	43,728^a^	1,125	0.89	0.40	0.45
OTU number	217^b^	225^b^	268^a^	228^b^	224^b^	201^b^	6.15	<0.01	0.14	<0.01
Ace	284^ab^	291^a^	293^a^	277^ab^	273^ab^	243^b^	6.31	0.04	0.19	0.14
Chao	271^c^	296^ab^	310^a^	248^d^	274^bc^	244^d^	7.29	<0.01	0.15	0.43
Shannon	2.29^b^	2.63^b^	3.17^a^	2.30^b^	2.49^b^	2.00^c^	0.10	<0.01	0.35	0.75
Simpson	0.13^bc^	0.15^bc^	0.11^c^	0.15^bc^	0.18^b^	0.25^a^	0.17	0.04	0.22	0.82
Coverage	99.9	99.9	99.9	99.9	99.9	99.9	-	-	-	-

SEM, standard error of the means; OTU, operational taxonomic units.

1) Corn and hairy vetch were mixed at proportions of 10:0 (Mix 10:0), 8:2 (Mix 8:2), 6:4 (Mix 6:4), 4:6 (Mix 4:6), 2:8 (Mix 2:8) and 0:10 (Mix 0:10) on a fresh weight basis, respectively.

2) T, effect of treatment; L, linear effect of treatment; Q, quadratic effect of treatment.

a–d Means in the same row with different letters are significantly different (p<0.05).
